# Midline-1 regulates effector T cell motility in experimental autoimmune encephalomyelitis via mTOR/microtubule pathway

**DOI:** 10.7150/thno.87130

**Published:** 2024-01-20

**Authors:** Yingying Wei, Wenjuan Li, Jie Huang, Zachary Braunstein, Xinxin Liu, Xinlu Li, Jeffrey Deiuliis, Jun Chen, Xinwen Min, Handong Yang, Quan Gong, Leya He, Zheng Liu, Lingli Dong, Jixin Zhong

**Affiliations:** 1 Department of Rheumatology and Immunology, Tongji Hospital, Huazhong University of Science and Technology, Wuhan, Hubei 430030, China.; 2 Cardiovascular Research Institute, Case Western Reserve University, Cleveland, Ohio 44106, USA.; 3 Wexner Medical Center, The Ohio State University, Columbus, Ohio 43210, USA.; 4 Sinopharm Dongfeng General Hospital, Hubei University of Medicine, Hubei Key Laboratory of Wudang Local Chinese Medicine Research (Hubei University of Medicine), Shiyan, Hubei 442008, China.; 5 Department of Immunology, School of Medicine, Yangtze University, Jingzhou, Hubei 434023, China.; 6 Department of Gastrointestinal Surgery, Tongji Hospital, Huazhong University of Science and Technology, Wuhan, Hubei 430030, China.; 7 Institute of Allergy and Clinical Immunology, Tongji Hospital, Tongji Medical College, Huazhong University of Science and Technology, Wuhan, Hubei 430030, China.; 8 Department of Otolaryngology-Head and Neck Surgery, Tongji Hospital, Tongji Medical College, Huazhong University of Science and Technology, Wuhan, Hubei 430030, China.; 9 Key Laboratory of Vascular Aging (HUST), Ministry of Education, Wuhan, Hubei 430030, China.

**Keywords:** Mid1, Experimental autoimmune encephalomyelitis, T cell migration, mTOR, Motility.

## Abstract

**Background:** Effector T cell activation, migration, and proinflammatory cytokine production are crucial steps in autoimmune disorders such as multiple sclerosis (MS). While several therapeutic approaches targeting T cell activation and proinflammatory cytokines have been developed for the treatment of autoimmune diseases, there are no therapeutic agents targeting the migration of effector T cells, largely due to our limited understanding of regulatory mechanisms of T cell migration in autoimmune disease. Here we reported that midline-1 (Mid1) is a key regulator of effector T cell migration in experimental autoimmune encephalomyelitis (EAE), a widely used animal model of MS.

**Methods:** *Mid1^-/-^* mice were generated by Crispr-Cas9 technology. T cell-specific Mid1 knockout chimeric mice were generated by adoptive transfer of *Mid1^-/-^* T cells into lymphocyte deficient *Rag2^-/-^* mice. Mice were either immunized with MOG_35-55_ (active EAE) or received adoptive transfer of pathogenic T cells (passive EAE) to induce EAE. *In vitro* Transwell^®^ assay or* in vivo* footpad injection were used to assess the migration of T cells.

**Results:** Mid1 was significantly increased in the spinal cord of wild-type (*Wt*) EAE mice and disruption of Mid1 in T cells markedly suppressed the development of both active and passive EAE. Transcriptomic and flow cytometric analyses revealed a marked reduction in effector T cell number in the central nervous system of *Mid1^-/-^* mice after EAE induction. Conversely, an increase in the number of T cells was observed in the draining lymph nodes of *Mid1^-/-^* mice. Mice that were adoptively transferred with pathogenic *Mid1^-/-^* T cells also exhibited milder symptoms of EAE, along with a lower T cell count in the spinal cord. Additionally, disruption of Mid1 significantly inhibited T-cell migration both *in vivo* and *in vitro*. RNA sequencing suggests a suppression in multiple inflammatory pathways in *Mid1^-/-^* mice, including mTOR signaling that plays a critical role in cell migration. Subsequent experiments confirmed the interaction between Mid1 and mTOR. Suppression of mTOR with rapamycin or microtubule spindle formation with colcemid blunted the regulatory effect of Mid1 on T cell migration. In addition, mTOR agonists MHY1485 and 3BDO restored the migratory deficit caused by Mid1 depletion.

**Conclusion:** Our data suggests that Mid1 regulates effector T cell migration to the central nervous system via mTOR/microtubule pathway in EAE, and thus may serve as a potential therapeutic target for the treatment of MS.

## Introduction

Multiple sclerosis (MS) is an autoimmune demyelinating disorder that primarily affects the central nervous system (CNS), leading to general paralysis in severe cases 1, 2. Although clinical treatments such as corticosteroids, monoclonal antibodies such as natalizumab, ocrelizumab, and alemtuzumab, sphingosine 1-phosphate receptor modulators, glatiramer acetate, and interferon are commonly used, the therapeutic effects of these treatments remain unsatisfactory 3. Experimental autoimmune encephalomyelitis (EAE) is a classic demyelinating mouse model that mimics MS in humans 4, 5. It is well recognized that inflammation-induced neural damage in EAE is mainly mediated by the effector T cells including type 1 (T_H_1) and type 17 (T_H_17) helper T cells, which are activated by myelin-presenting innate immune cells in the draining lymph nodes (dLNs). The activated T cells then migrate to the CNS, leading to central inflammatory infiltration and axonal degeneration 6. T_H_1 and T_H_17, two important subtypes of CD4^+^ T cells, are generally accepted as the major pathogenic effector cells in EAE 6 and their transendothelial migration is a critical process in the pathogenesis 7. The interaction between α4/β1 integrins on T cells and type I vascular cell adhesion proteins (VCAM-1) on endothelial cells is associated with early entry of T cells into the CNS. In addition, chemokines including CXCL16 and CXCL12 are increased in CNS inflammation 8, 9. This T cell migration has been shown as a promising therapeutic target for MS. Intercepting of α4 integrins prevents encephalitic T cells from infiltration to the CNS and remits the pathogenesis of EAE 10-12. Natalizumab treatment targeting α4 integrins may reduce the risk of progressive disability and clinical recurrence in patients with recurrent MS 13. Naringenin downregulates chemokine receptor CCR7 on CD4^+^ T cells in the CNS, reducing pathogenic T cell migration into the CNS and decreasing the severity of EAE 14. The blockade of chemokine receptor CXCR3 can also inhibit T cell migration into the CNS, thereby mitigating the development of passive EAE 15. However, the exact mechanisms regulating pathogenic T cell migration in MS remain elusive, hampering the development of effective therapeutic approaches targeting this migration.

Midline-1 (Mid1) is a microtubule-binding molecule involved in embryonic development and an E3 ubiquitin ligase belonging to the triple-motif (TRIM) family 16, 17. Loss-of-function mutations of Mid1 cause Opitz G/BBB syndrome in humans. Additionally, it has been reported to have involvement in the pathology of asthma, cancer, and neurodegenerative diseases 18-20. However, its implication in autoimmune disease has not been extensively studied 21, 22. A study reported that Mid1 is highly expressed in murine killer cells and controls their degranulation process 23. We recently showed that Mid1 mediates the promotive effect of dipeptidyl peptidase-4 on T cell migration and accelerates atherosclerosis in mice 24. These studies indicate that Mid1 may play a key role in regulating T cell inflammation. Nevertheless, the role of Mid1 in EAE has not been explored. Here, we examined the impact of Mid1 on EAE by using Mid1 knock-out mice and adoptive transfer of pathogenic T cells. In addition, we investigated the pathway through which Mid1 regulates T cell migration in the scenario of EAE.

## Materials and Methods

### Mice

*Mid1^-/-^* mice were generated using CRISPR-Cas9 technology as we previously described 24. *Rag2^-/-^* and *CD45.1* mice were purchased from Shanghai Model Organisms Center, Inc. All the mice used in the experiment were kept in the SPF animal facilities at Tongji Hospital. All experiments reported herein were approved by the Institutional Animal Care and Use Committee of Tongji Hospital and conducted following the animal use guidelines of the institute.

### Induction and evaluation of active EAE

For the construction of chimeric mice, *Rag2^-/-^* mice were adoptively transferred with 10^7^ wild-type (*Wt*) T cells, *Mid1^-/-^* T cells, or a mixture of CD45.1-expressing* Wt* and CD45.2-expressing *Mid1^-/-^* T cells at a 1:1 ratio. *Wt*, *Mid1^-/-^*, or chimeric mice were then used for the induction of active EAE as previously reported 25. Briefly, mice were subcutaneously injected with a 200 μL emulsion that contains 1 mg/ml MOG_35-55_ peptide (MEVGWYRSPFSRVVHLYRNGK), 2.5 mg/ml Mycobacteria tuberculosis H37Ra (B.D.), and 100 μL Freund's adjuvant (Sigma). On the day of immunization and two days after immunization, mice were intraperitoneally injected with 200 ng of pertussis toxin (List Biological Laboratories Inc.).

### Statistical analysis

All data are presented as mean ± standard error of the mean (SEM). The difference in EAE clinical score was assessed with two-way ANOVA. EAE incidence was compared with χ^2^ test using GraphPad Prism 8. The differences between the means of the two groups were accomplished by Student's t-test. The differences between the means of 3 or more independent groups were accomplished by one-way ANOVA. Using GraphPad Prism 8 for statistical analysis and graphing, *p* < 0.05 was considered statistically significant.

Please refer to the supplementary data for detailed materials and methods

## Results

### Deficiency of Mid1 protects mice from MOG_35-55_ induced EAE

**Figure 1:**
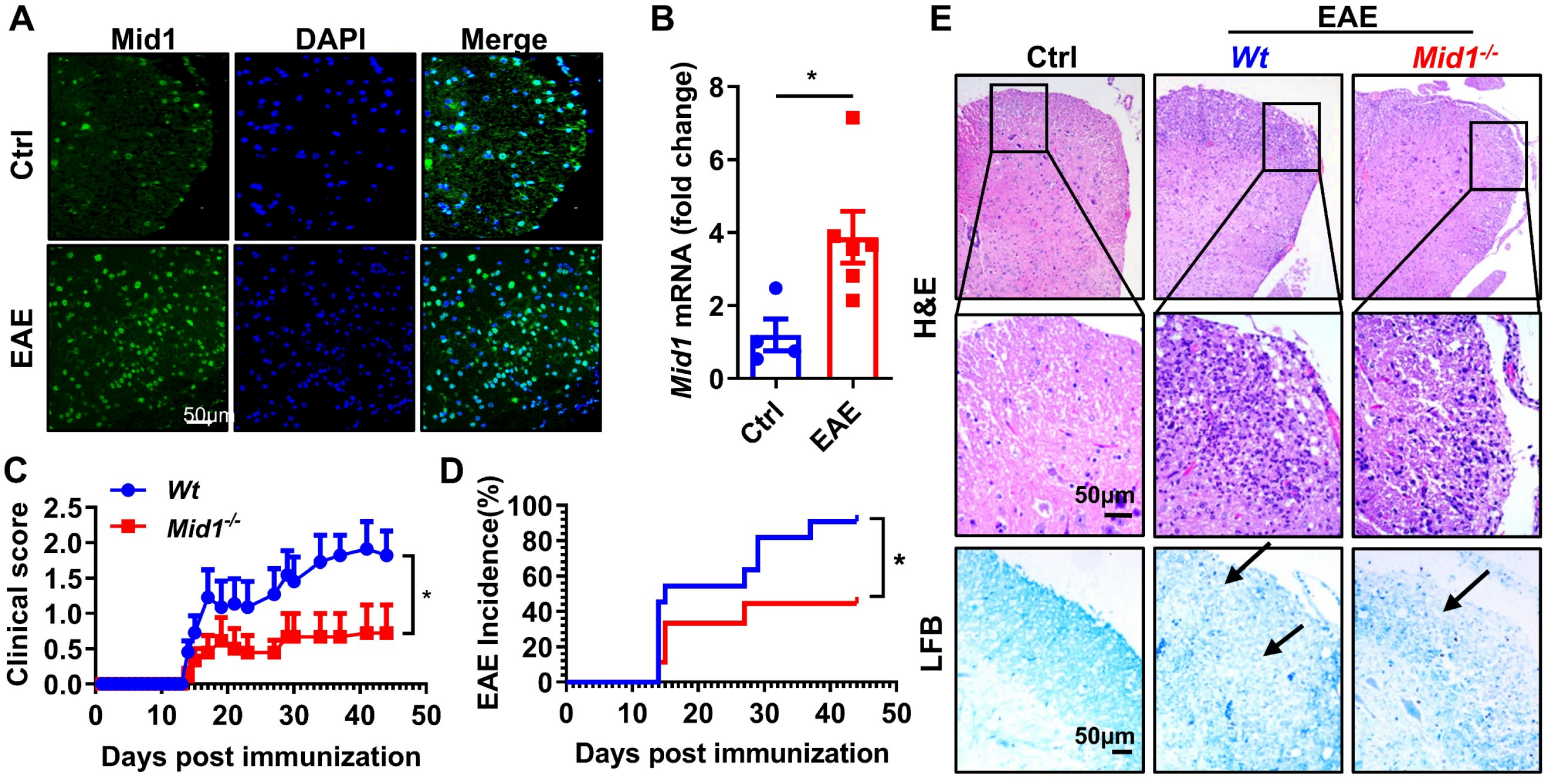
**Deficiency of Mid1 protects mice from MOG_35-55_ induced EAE. A.** Eight-week-old *Wt* mice were randomly divided into two groups. For the EAE group, one set of mice was subcutaneously injected with emulsified MOG_35-55_ to establish an EAE model on day 0. Pertussis toxin (PTX) was administered intraperitoneally on day 0 and day 2. For the control (Ctrl) group, the animals were untreated and served as the control for EAE. After immunization, spinal cord tissue was used for tissue immunofluorescence staining of Mid1. Left, Mid1; middle, DAPI; right, merged image. **B.** Tissues were harvested 30 days after the disease onset and RNA was extracted from the spinal cord tissues of control or EAE mice, and the mRNA level of *Mid1* was measured by real-time PCR. *, p < 0.05. **C-D.** Eight-week-old *Wt/Mid1^-/-^* mice were immunized with MOG_35-55_ peptides and monitored for EAE clinical symptom scores and incidence. Data are representatives of three independent experiments. Data are shown as mean ± SEM. *p < 0.05. **E.** Spinal cord of control untreated *Wt* mice, immunized *Wt*, and immunized *Mid1^-/-^* EAE mice was used for Haematoxylin-Eosin (H&E) staining and Luxol Fast Blue (LFB) staining.

In MOG_35-55_-immunized EAE mice, the expression of Mid1 in the spinal cord was significantly elevated when compared to unimmunized *Wt* mice, as evidenced by both real-time quantitative PCR and immunofluorescence staining (**Figures 1A-B**). To examine if Mid1 is implicated in the pathogenesis of EAE, we induced active EAE in *Wt* and *Mid1* knockout mice. As depicted in **Figures 1C-E**, *Mid1^-/-^* mice were much more resistant to EAE induction, as manifested by improved disease symptom score, reduced EAE incidence, reduced inflammatory cell infiltration, and lower levels of demyelination. The incidence of EAE was also reduced in *Mid1^-/-^* group (90.9% in *Wt* vs. 44.4% in *Mid1^-/-^*, p < 0.05). The average EAE symptom score was also markedly lower in *Mid1^-/-^* group when compared to the *Wt* group. Histological examination showed significantly improved inflammatory cell infiltration and demyelination in the spinal cord of *Mid1^-/-^* mice after MOG_35-55_-immunization (**Figure 1E**).

### Transcriptomic profile indicates a reduced expression of T cell-related genes in *Mid1^-/-^* mice after EAE induction

**Figure 2:**
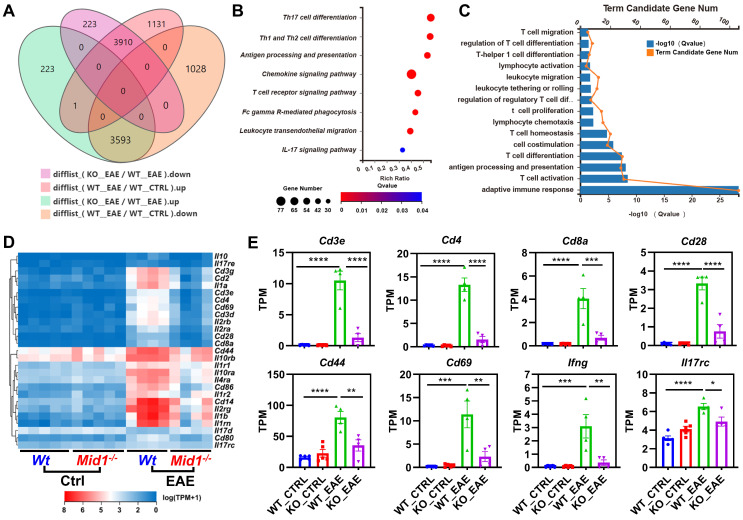
**CNS Transcriptomic profile of *Wt* and *Mid1^-/-^* EAE mice. A.** RNA-seq analysis was performed using the spinal cord tissues collected from untreated or MOG_35-55_-immunized *Wt* and *Mid1^-/-^* mice (n = 5 for untreated and n = 4 for EAE mice). Venn diagram shows the number of genes up-regulated or down-regulated in KO_EAE *vs*. WT_EAE and/or WT_EAE *vs*. WT_Ctrl. **B.** KEGG pathway analysis was performed using 3910 down-regulated differentially expressed genes, showing that most of the enriched pathways are related to T cell response. **C.** GO process analysis using 4242 down-regulated differentially expressed genes shows that most of the enriched pathways are related to T cell response. **D.** Heat map showing representative genes that are critical for T cells. **E.** Bar graphs showing representative marker genes for T cells. Data are shown as mean ± SEM. *, p < 0.05; **, p < 0.01; ***, p < 0.001; ****, p < 0.0001.

To characterize the transcriptomic profile of *Mid1^-/-^* mice, we performed RNA sequencing on spinal cords isolated from untreated or MOG_35-55_-immunized *Wt* and *Mid1^-/-^* mice. Principal component analysis (PCA) analysis indicates distinct mRNA expression profiles among the 4 groups (**Figure S1A**). We identified 7504 EAE-related genes in the differential expression genes between the groups KO_EAE and WT_EAE, 3910 of which were downregulated in the KO_EAE group when compared to WT_EAE and upregulated in WT_EAE when compared to WT_Ctrl (**Figure 2A, Figure S1B**). Nevertheless, there were minimal differences between *Wt* control and *Mid1^-/-^* control groups (**Figure S1C**), suggesting that Mid1 deficiency may not cause significant abnormalities under physiologic conditions. We subsequently performed KEGG pathway and GO enrichment analyses of genes that were diminished in the KO_EAE group (**Figures 2B-C**). The majority of the differentially expressed genes were enriched in T cell differentiation- and migration-related pathways. Clustering analysis further discovered a notable reduction in the marker genes of T cells and T cell activation, such as *Cd3, Cd4, Cd8, Ifng, Il17rc, Cd44,* and *Cd69* (**Figures 2D-E**). In addition, GSEA enrichment analysis suggests a downregulation of T cell migration- and activation-related pathways in *Mid1^-/-^* mice after EAE onset (**Figure S2**).

### Loss of Mid1 suppresses effector T cell infiltration in the CNS after EAE induction

**Figure 3:**
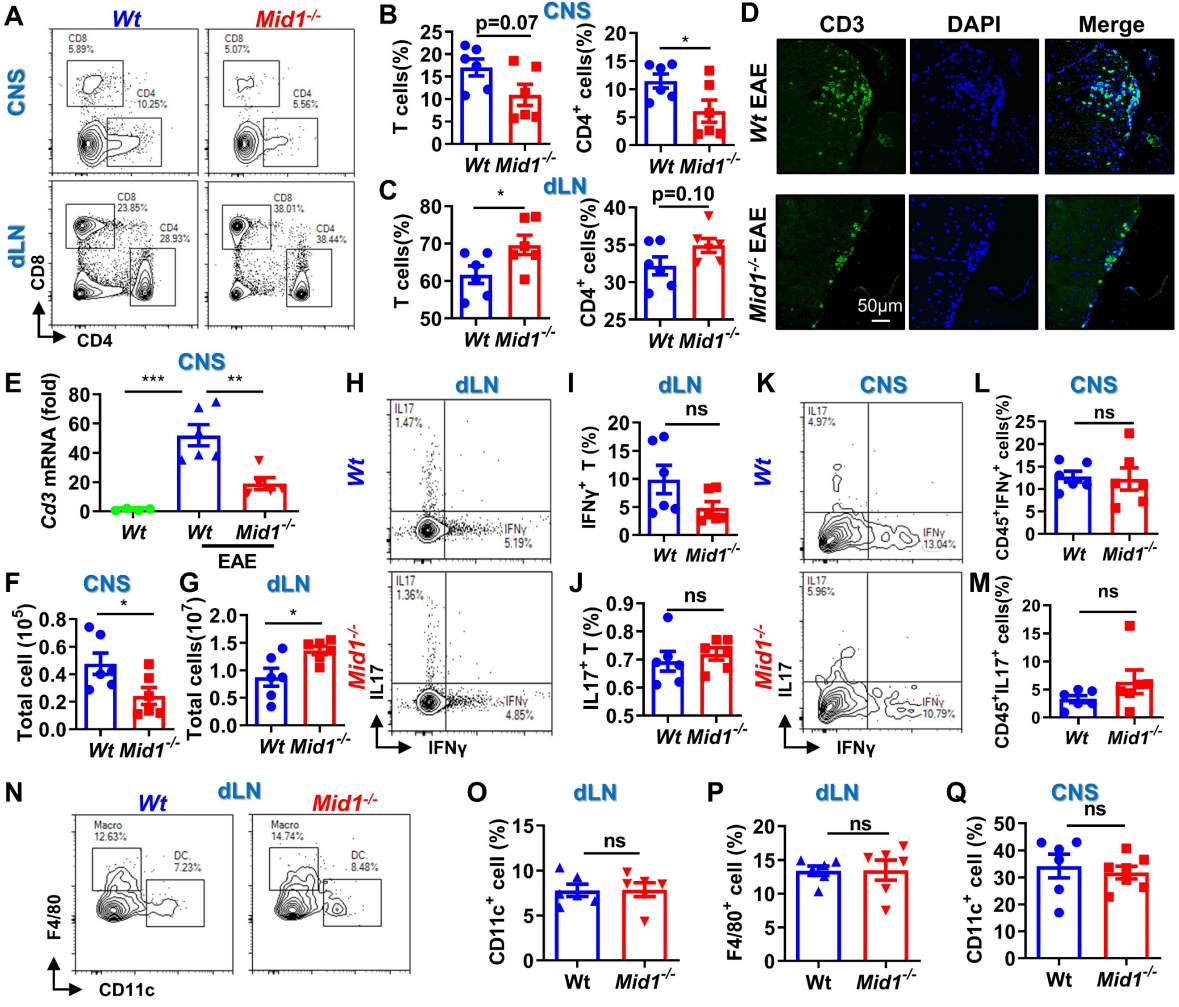
**Loss of Mid1 suppresses effector T cell infiltration in the CNS after EAE induction. A-C,** Eight-week-old *Wt* and *Mid1^-/-^* mice were immunized with MOG_35-55_ peptides to induce EAE (n = 6). Spinal cord and draining lymph nodes (dLN) were harvested for the isolation of single cell suspension 30 days after the immunization. Cells were then stained with anti-mouse CD4 and CD8 antibodies, followed by flow cytometric detection of T cells. Representative density plots (**A**) and statistical analysis (**B-C**) showed the percentages of CD4^+^ and CD8^+^ cells in the spinal cord (CNS) and dLN. Data are shown as mean ± SEM. **C.** Bar chart of the proportion of T cells and CD4^+^ T cells in the dLN of *Wt/Mid1^-/-^* mice. Data are shown as mean ± SEM. *, p < 0.05. **D,** Spinal cord sections from the mice treated as above were used for tissue immunofluorescence staining (IF) of CD3 (green fluorescence) and nucleus (blue, DAPI staining).** E,** Total mRNA was extracted from the spinal cord tissues of EAE mice and unimmunized *Wt* control mice. The transcription level of *Cd3* was measured by real-time PCR. Data are shown as mean ± SEM. **, p < 0.01; ***, p < 0.001. **F-G,** Total cell numbers were counted for single cell suspensions isolated from the spinal cord (**F**) and dLN (**G**) of EAE mice. Data are shown as mean ± SEM. *, p < 0.05. **H,** Cells isolated from the dLN were stained with anti-mouse CD3 antibodies, followed by intracellular staining of IFNγ and IL17. This figure showed a representative density map of CD3^+^ IFNγ^+^, CD3^+^IL17^+^cells. **I-J,** Bar chart of the proportions of CD3^+^ IFNγ^+^(**I**) , CD3^+^IL17^+^(**J**) cells in the dLN of *Wt/Mid1^-/-^* mice. Data are shown as mean ± SEM. **K,** Representative dot plot of CD45^+^IFNγ^+^, CD45^+^IL17^+^ cells in the EAE mice spinal cord. **L-M,** Bar chart of the percentages of CD45^+^IFNγ^+^ (**L**) and CD45^+^IL17^+^ (**M**) cells in the spinal cord of *Wt/Mid1^-/-^* mice. Data are shown as mean ± SEM. **N-P,** Single cells from dLN were stained with anti-mouse CD11b, CD11c and F4/80 antibodies. Representative flow cytometric density plots (**N**) and statistical bar graphs showed the proportions of CD11c^+^ dendritic cells (**O**) and F4/80^+^ macrophages (**P**) in the dLN of MOG_35-55_-immunized *Wt* and *Mid1^-/-^* mice. Data are shown as mean ± SEM. Ns, not significant. **Q,** Bar graph showing the proportion of CD11c^+^ dendritic cells in the spinal cord of *Wt/Mid1^-/-^* EAE mice. Data are shown as mean ± SEM. Ns, not significant. All figures are representatives of three independent experiments.

Since RNA sequencing data revealed a significant downregulation of T cell-related genes in *Mid1^-/-^* mice after EAE induction, we next used flow cytometry to detect the proportion of T cells in the spinal cord and dLN of *Wt* and *Mid1^-/-^* mice. As expected, the proportion of effector T cells in the dLN of *Wt* EAE mice were higher than those in the unimmunized *Wt* mice (**Figure S3A**). Meanwhile, MOG_35-55_-immunized mice showed reduced plasma levels of anti-inflammatory cytokine IL-10 and increased levels of the pro-inflammatory cytokines IFNγ, TNFα, and IL-6 (**Figure S3B**). Interestingly, T cells were markedly decreased in the CNS of *Mid1^-/-^* mice after EAE induction when compared to *Wt*, whereas T cell number was slightly higher in the dLN of *Mid1^-/-^* mice (**Figures 3A-C**). A decreased infiltration of CD3^+^ cells in the CNS of *Mid1^-/-^* mice was further observed by immunofluorescence (**Figure 3D**). Additionally, real-time PCR also confirmed a significant decline of the T cell marker *Cd3* in the CNS of *Mid1^-/-^* mice after EAE induction (**Figure 3E**). Consistent with these findings, the absolute number of total inflammatory cells was decreased in the spinal cord and increased in the dLN of *Mid1^-/-^* mice, when compared to *Wt* (**Figures 3F-G**). We next examined if deficiency of Mid1 affects the differentiation of IFNγ-producing (type 1) and IL17-producing (type 17) effector T cells. The percentages of CD3^+^IFNγ^+^ and CD3^+^IL17^+^ effector T cells in both spinal cord and dLN were similar in *Mid1^-/-^* and *Wt* mice (**Figures 3H-M**).

There were no significant differences between *Wt* and *Mid1^-/-^* in the proportions of dendritic cells (DCs), macrophages, and CD11b^+^ cells in CNS tissue and dLN (**Figures 3N-Q, Figure S4**). This data suggests that Mid1 deficiency reduced the infiltrations of T cells, without affecting T cell polarization and infiltration of DCs/macrophages. The reduction of T cell infiltration in the CNS of* Mid1^-/-^* mice was also confirmed in the acute phase, 1 week after the onset of EAE (**Figure S5**).

### Mid1 deficiency suppresses CNS inflammation and passive EAE

**Figure 4:**
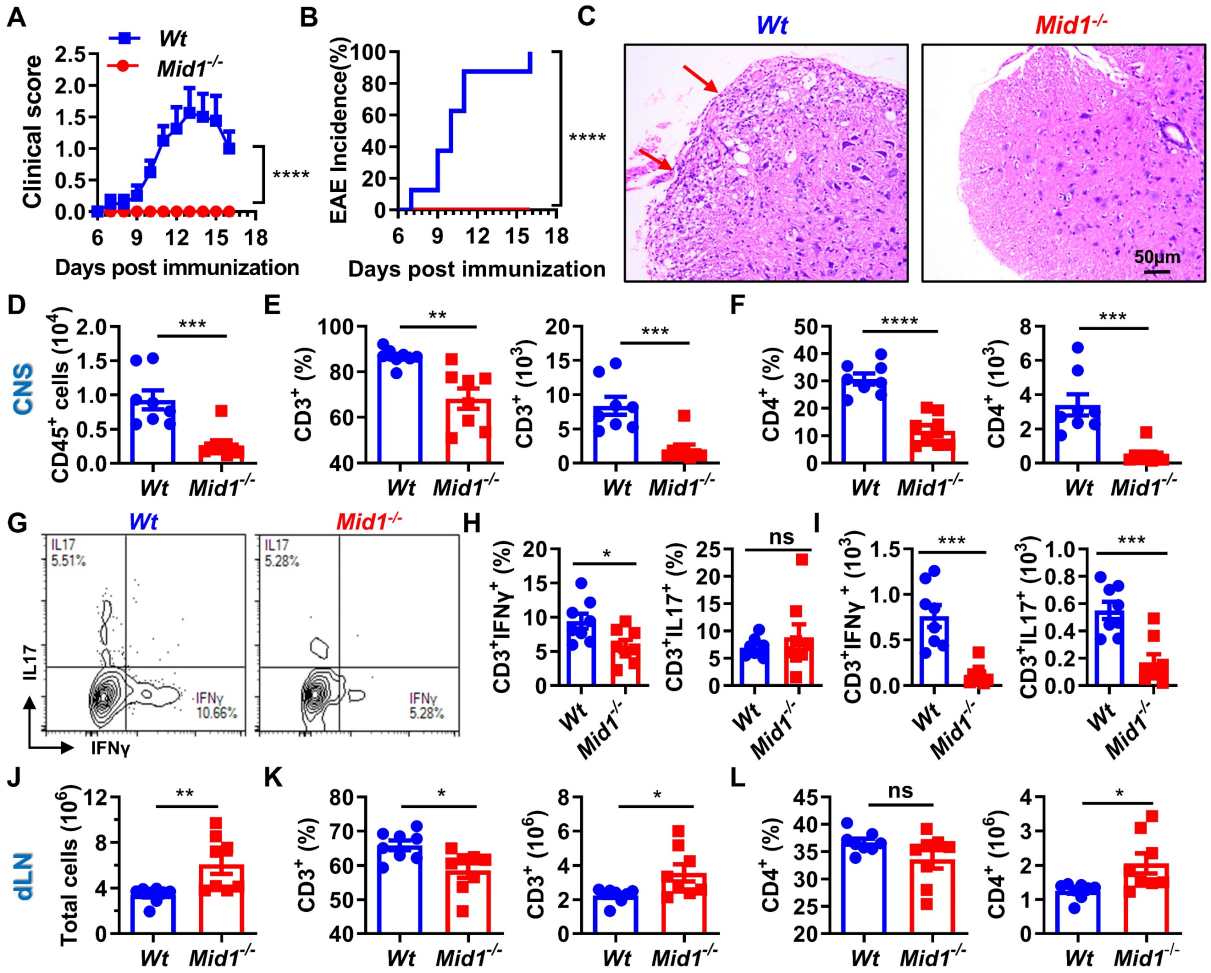
**Midline-1 knockout reduced the severity of passive EAE. A-B,** Eight-week-old *Wt* and *Mid1^-/-^* mice were immunized with MOG_35-55_ to induce active EAE. *Wt* and *Mid1^-/-^* mice with comparable disease scores were sacrificed on day 16 after immunization, and the spleen and lymph nodes were used to prepare single-cell suspension. Cells at the concentration of 10^7^/mL were then incubated with 5 ng/mL IL-2 and 20 µM MOG_35-55_ for 48 h at 37°C, followed by adoptive transfer into *Wt* recipient mice through the canthal vein. EAE symptom score (**A**) and incidence (**B**) were shown. Data are shown as mean ± SEM. ***, p < 0.001; ****, p < 0.0001.**C,** Spinal cord sections of mice receiving *Wt* and *Mid1^-/-^* pathogenic cells were used for H&E staining. Red arrows indicate inflammatory infiltration. **D-F,** Single cell suspension was prepared from the spinal cord of animals with passive transfer of *Wt* and *Mid1^-/-^* pathogenic cells, followed by staining with anti-mouse CD45, CD3, and CD4. The proportions and numbers of CD45^+^, CD3^+^, and CD4^+^ cells are shown. Data are shown as mean ± SEM. **, p < 0.01; ***, p < 0.001; ****, p < 0.0001. **G.** Single cells from spinal cord were stained with anti-mouse CD3 antibodies, followed by intracellular staining of IFNγ and IL17. Representative density plots showed the frequencies of CD3^+^ IFNγ^+^ and CD3^+^IL17^+^cells. **H-I,** Bar graphs showed the proportion and number of CD3^+^ IFNγ^+^ and CD3^+^IL17^+^cells in the spinal cord of *Wt* and *Mid1^-/-^* mice. Data are shown as mean ± SEM. *, p < 0.05; ***, p < 00.01; ns, not significant. **J.** Total cells from the dLN of EAE mice were counted. Data are shown as mean ± SEM. **, p < 0.01. **K-L,** Single cells isolated from the dLN were stained with anti-mouse CD3 and CD4. The proportions and numbers of CD3^+^ and CD4^+^ cells were shown. Data are shown as mean ± SEM. *, p < 0.05. All figures are representative of two independent experiments.

After observing a reduced infiltration of T cells in the CNS and an increased number of T cells in the dLN of *Mid1^-/-^* mice after EAE induction, we speculated that Mid1 is required for T cell migration from dLN to the CNS. To test this hypothesis, we adoptively transferred pathogenic T cells isolated from the spleen and dLN of *Wt/Mid1^-/-^* EAE mice to *Wt* recipients. While transfer of *Wt* pathogenic T cells successfully induced EAE onset on *Wt* recipients, mice transferred with *Mid1^-/-^* pathogenic T cells failed to develop EAE (**Figures 4A-B**). Consistent with this, inflammatory infiltration was observed in the spinal cord of mice transferred with *Wt* pathogenic cells, but not with *Mid1^-/-^* pathogenic cells (**Figure 4C**). Flow cytometric detection of CNS-infiltrating cells confirmed the lower numbers of CD45+ leukocytes, total T cells (CD3^+^), CD4^+^ T cells, as well as IFNγ- and IL-17-producing T cells in the CNS of mice with *Mid1^-/-^* pathogenic cells when compared to those with *Wt* pathogenic cells (**Figures 4D-I**). In contrast, the immune cells within the DLN were increased in mice receiving *Mid1^-/-^* pathogenic cells when compared to those with *Wt* pathogenic cells (**Figures 4J-L**). These findings indicate that Mid1 deletion in T cells results in disrupted pathogenic cell infiltration in the CNS in EAE.

### The resistance to EAE depends on T cells

**Figure 5:**
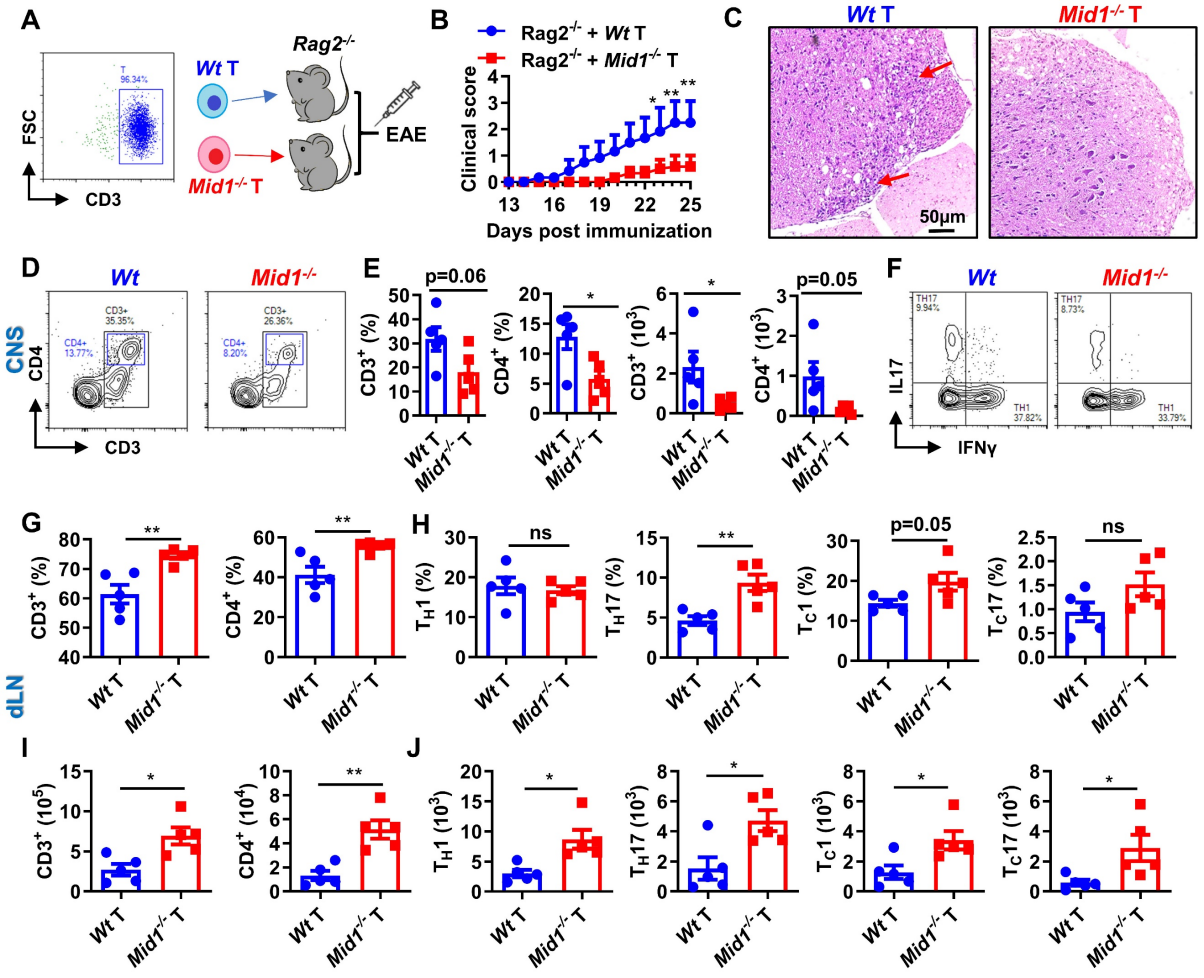
** Mid1 deficiency protects mice from EAE by T cells. A**, Flowchart showing the generation chimeric mice with *Wt* or *Mid1^-/-^* T cells. *Wt* and *Mid1^-/-^* T cells were enriched from *Wt* and *Mid1^-/-^* mice, and the purity was verified to be higher than 95% by flow cytometry. Purified *Wt* and *Mid1^-/-^* T cells were then adoptively transferred into *Rag2^-/-^* mice to generate chimeric animals, followed by EAE induction with MOG_35-55_ 2 days later. **B,** EAE clinical symptoms were monitored daily. Data are shown as mean ± SEM. *, p < 0.05; **, p < 0.01. **C,** Spinal cord sections of chimeric mice with *Wt* or *Mid1^-/-^* T cells were harvested 30 days after the immunization and used for H&E staining. Red arrows showing the areas of inflammatory infiltration. **D,** Spinal cord tissues were digested for the isolation of single cell suspension for flow cytometry. Single cells were stained with anti-mouse CD45, CD3, and CD4, Representative dot plots showing the gating of CD3^+^ and CD4^+^ T cells. **E,** The proportions and numbers of CD45^+^CD3^+^, CD45^+^CD3^+^CD4^+^ T cells were analyzed. Data are shown as mean ± SEM. *, p < 0.05. **F,** Single cells from the spinal cords were intracellularly stained with IFNγ and IL17 after cell surface staining with anti-mouse CD4 antibody. Representative figures show the frequencies of IFNγ^+^ and IL17^+^ populations in CD4^+^ gate. **G-I,** Single cells from the dLN were stained with anti-mouse CD3 and CD4. The proportions (**G**) and numbers (**I**) of CD3^+^ and CD4^+^ T cells were shown. Data are shown as mean ± SEM. *, p < 0.05; **, p < 0.01. **H-J**, Single cells from the dLN were intracellularly stained with IFNγ and IL17 after cell surface staining with CD4 and CD8. The proportions (**H**) and numbers (**J**) of CD4^+^IFNγ^+^ (T_H_1), CD8^+^IFNγ^+^ (T_C_1), CD4^+^IL17^+^ (T_H_17) and CD8^+^IL17^+^ (T_C_17) cells were shown. Data are shown as mean ± SEM. *, p < 0.05; **, p < 0.01; ns, not significant. All figures are representatives of three independent experiments.

Active and passive EAE experiments showed a redistribution of T cells, but not DCs or macrophages, in the CNS and dLN of *Mid1^-/-^* mice. To further verify if Mid1 deficiency-associated protection of EAE is dependent on T cells, we adoptively transferred *Wt* and *Mid1^-/-^* T cells into lymphocyte-deficient* Rag2^-/-^* mice to establish a chimeric mouse model with T cell-specific Mid1 deficiency (**Figure 5A**). Mice reconstituted with *Mid1^-/-^* T cells are more resistant to EAE compared to those with *Wt* T cells, as manifested by lower EAE symptom score and less spinal cord inflammatory infiltration (**Figures 5B-C**). In addition, we found that 3 out of the 8 *Rag2^-/-^* mice with *Wt* T cells died after EAE induction, while all the 5 *Rag2^-/-^* mice with* Mid1^-/-^* T cells survived (**Figure S6**). Similarly, we found T cell-specific deletion of Mid1 results in a significant reduction in the proportion and absolute number of CD3^+^ T cells and CD4^+^ T cells in the spinal cord upon MOG_35-55_ challenge, without affecting T_H_1 and T_H_17 differentiation (**Figures 5D-F**). In contrast to the CNS, the proportions and numbers of CD3^+^ and CD4^+^ T cells, as well as their IFNγ-producing type 1 and IL17-producing type 17 subpopulations were significantly elevated in the dLN of chimeric mice with* Mid1^-/-^* T cells (**Figures 5G-J**). This data suggests that the protective effect of Mid1 deficiency on EAE is dependent on T cells.

### Defective of Mid1 inhibits T cell migration

**Figure 6:**
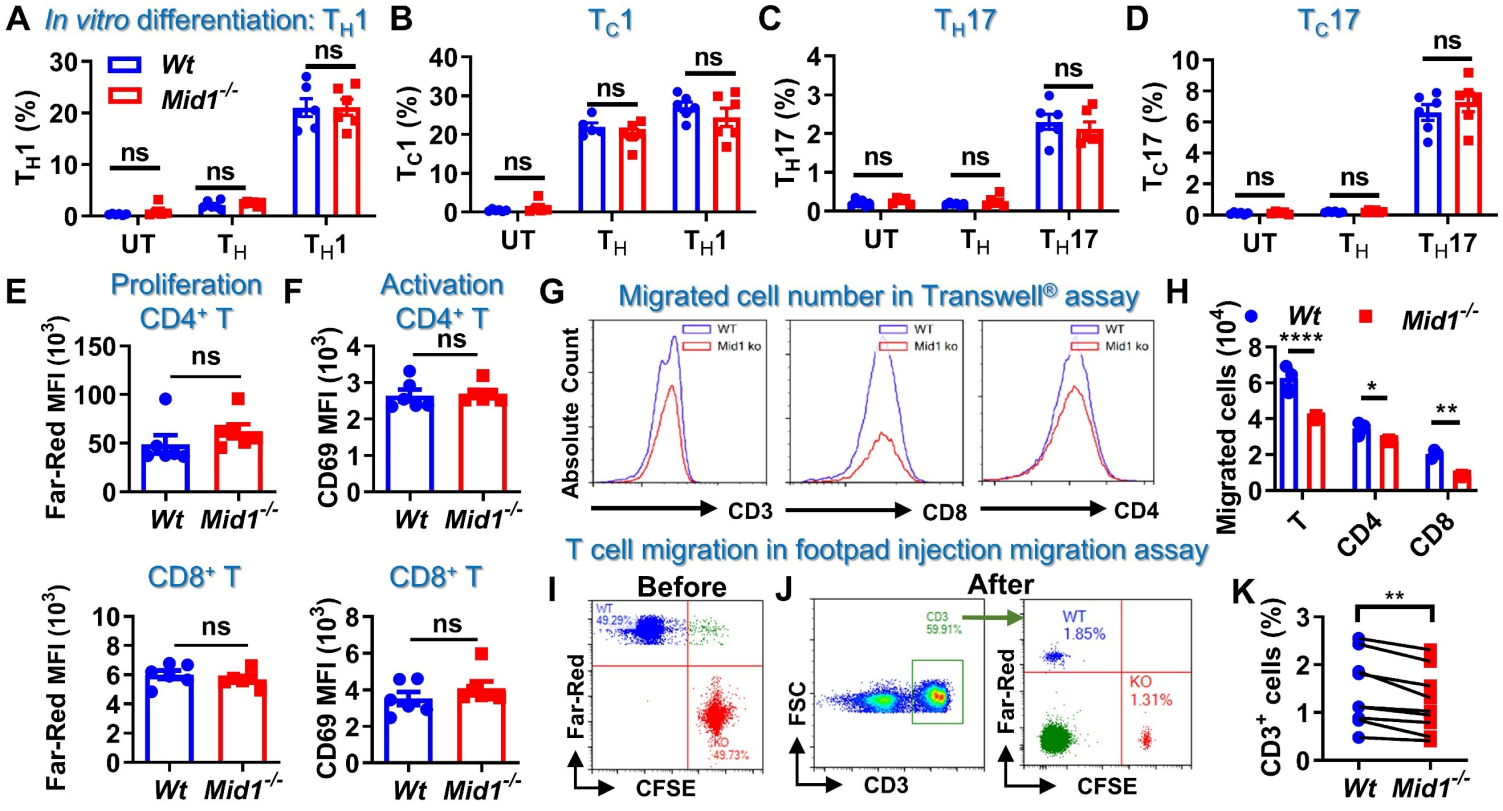
** Defective of Midline-1 inhibits T-cell migration *in vitro*. A-D,** Splenocytes from *Wt* and *Mid1^-/-^* mice were cultivated for 72 h at 37 °C under the differentiation conditions of untreated (UT), T_H_ (anti-CD3, anti-CD28), T_H_1 (anti-CD3, anti-CD28, IL-12), and T_H_17 (anti-CD3, anti-CD28, TGFβ, IL-6) conditions, followed by surface staining with CD4 and CD8 as well as intracellular staining with IFNγ and IL17. The proportions of T_H_1 (CD4^+^ IFNγ^+^), T_H_17 (CD4^+^ IL17^+^), T_C_1 (CD8^+^ IFNγ^+^), and T_C_17 (CD8^+^ IL17^+^) were determined. Data are shown as mean ± SEM. ns, not significant.** E-F,** Splenocytes from *Wt* and *Mid1^-/-^* mice were labeled with CellTrace™ Far-Red. After stimulation with anti-CD3 and anti-CD28 antibodies at 37 °C for 72 h, cells were then stained with CD4, CD8 and T cell activation marker CD69. Flow cytometry was performed to detect the mean fluorescence intensity (MFI) Far-Red and CD69 in CD4^+^ and CD8^+^ cells to examine the proliferation and activation respectively.** G-H,** Splenocytes from *Wt* and *Mid1^-/-^* mice were added to the insert of a 24-well Transwell^®^ plate, with the lower chamber filling with 1640 medium containing 400 ng/mL CCL-19. After 6 h of incubation at 37°C, the cells migrated to the lower chamber were counted and harvested for flow cytometric detection of CD3, CD4, and CD8. Representative histograms (**G**) and statistical analysis (**H**) showed the migration of *Wt* and *Mid1^-/-^* CD3^+^, CD4^+^, and CD8^+^ T cells. *, p < 0.05; **, p < 0.01; ****, p < 0.0001. **I-K,** Splenocytes from *Wt* and *Mid1^-/-^* mice were fluorescently labeled with CellTrace™ Far-Red or CellTrace™ CFSE and mixed with equal proportion (**I**). Mixed cells were then subcutaneously injected into the footpad of *Wt* mice. After 12 h, the popliteal lymph nodes were isolated and the fractions of Far-Red-labeled* Wt* and CFSE-labeled* Mid1^-/-^* CD3^+^ T cells were measured using flow cytometry (**J-K**). **, p < 0.01. All figures are representatives of three independent experiments.

T cell differentiation, proliferation, and migration are key steps regulating the pathogenesis of EAE 26, 27. It was found that Mid1 knockout did not affect the proportions of CD4^+^ and CD8^+^ T cells, as well as their type 1 and type 17 subsets in untreated mice (**Figures S7-S8**). Mid1 deficiency also had no significant impact on effector T cell proliferation, differentiation, or activation in *in vitro* conditions (**Figures 6A-F**). As depicted above, *Wt* EAE mice had a greater percentage and quantity of effector T cells in CNS tissue and dLN than unimmunized mice, while the elevation of T cells was suppressed in CNS tissue, but not dLN, in *Mid1^-/-^* mice immunized with MOG_35-55_. This suggests that the migration of T cells from dLN to the CNS might be impaired in *Mid1^-/-^* mice. Therefore, we examined the migratory activity of *Mid1^-/-^* T cells using Transwell^®^ migration assay. There were fewer *Mid1^-/-^* T cells migrated to the lower chamber when compared to *Wt* (**Figures 6G-H, & S8**). Next, CellTrace™ Far-Red-labeled *Wt* splenocytes and CFSE-labeled *Mid1^-/-^* splenocytes were mixed in equal numbers and then injected into the footpad of *Wt* mice (**Figure 6I**). The number of CFSE-labeled *Mid1^-/-^* T cells was less than Far-red-labeled *Wt* T cells in the popliteal lymph nodes 12 h after the injection, indicating a reduced migratory activity in *Mid1^-/-^* T cells (**Figures 6J-K**).

### Disruption of Mid1 inhibits T cell migration in EAE

**Figure 7:**
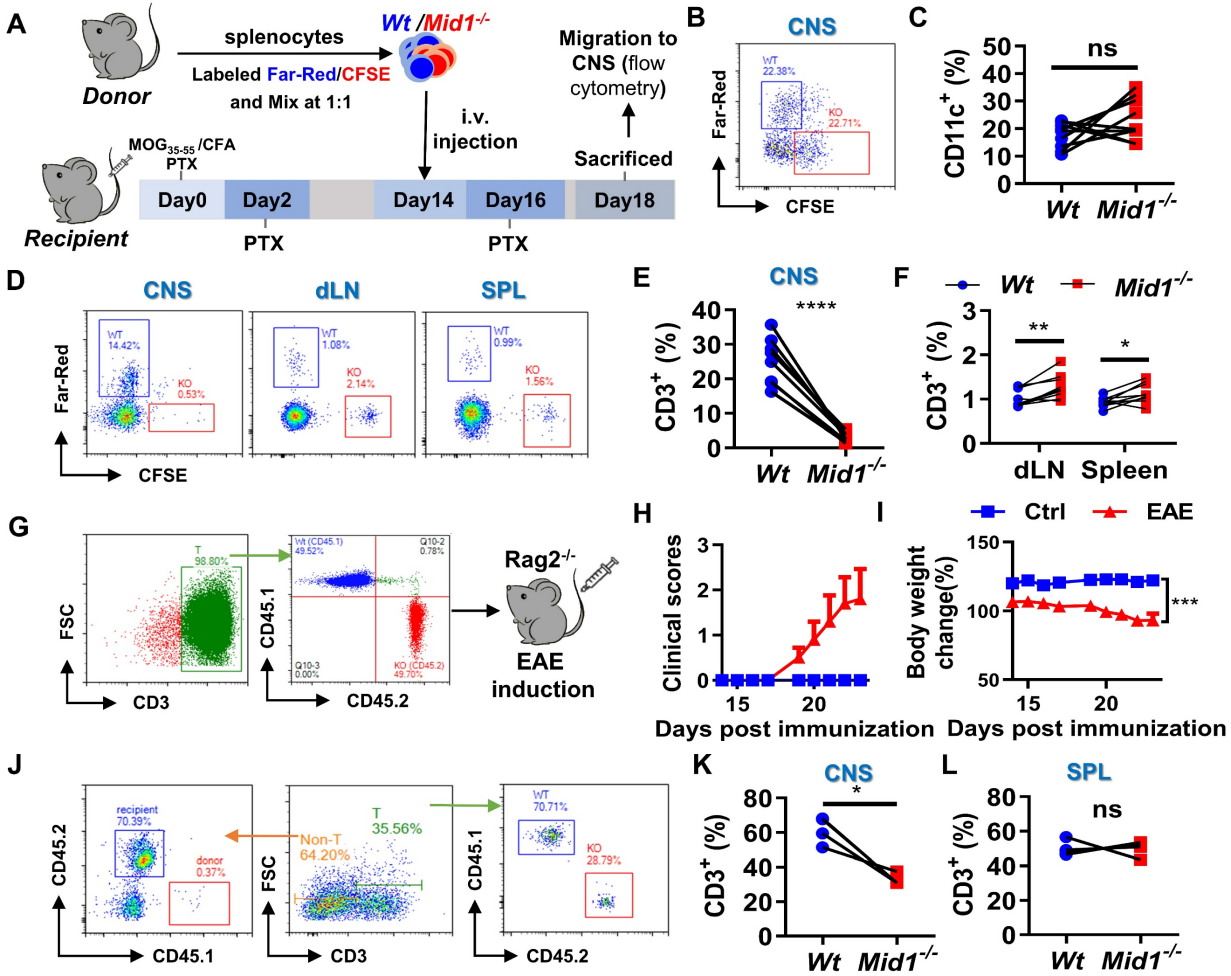
**Defective of Midline-1 inhibits T-cell migration to alleviate EAE. A-F**, Flowchart showing the generation chimeric mice with *Wt* or *Mid1^-/-^* splenocytes. Splenocytes from *Wt* and *Mid1^-/-^* mice were labeled with Far-Red and CFSE, respectively. Labeled cells were mixed at 1:1 ratio and then adoptively transferred into *Wt* EAE mice via angular vein on day 14 of immunization, followed by PTX injection on day 16 (**A**). After 96 h of adoptive transfer, the recipient mice were sacrificed and the fractions of Far-Red-labeled* Wt* and CFSE-labeled* Mid1^-/-^*CD11C^+^ DCs and CD3^+^ T cells were determined in the spinal cord, dLN, and spleens by flow cytometry. Representative density plots (**B**) and statistical graph (**C**) of CD11c^+^ DCs show similar tissue distributions of *Wt* and *Mid1^-/-^* DCs. Representative density plots (**D**) and statistical graphs (**E-F**) showing the proportions of *Wt* and *Mid1^-/-^* T cells indicate a differential distribution of *Wt* and *Mid1^-/-^* T cells in the CNS and peripheral tissues. *, p < 0.05; **, p < 0.01; ***, p < 0.001; ns, not significant.** G-L,** CD45.1-expressing *Wt* T cells and CD45.2-expressing *Mid1^-/-^* T cells were purified and mixed at a ratio of 1:1. Mixed cells were then adoptively transferred to *Rag2^-/-^* mice, followed by immunization with MOG_35-55_. The flowchart (**G**), EAE disease scores (**H**) and body weight change of mice (**I**) were shown. Single-cell suspensions isolated from the CNS and spleen were stained with anti-mouse CD45.1, CD45.2 and CD3 antibodies and submitted to flow cytometric detection. Representative density plots (**J**) showed gating strategy and percentages of CD45.1-expressing *Wt* T cells and CD45.2-expressing *Mid1^-/-^* T cells in the CNS. Statistical graphs showed the proportions of CD45.1-expressing *Wt* T cells and CD45.2-expressing *Mid1^-/-^* T cells in the CNS (**K**) and spleen (**L**). *, p < 0.05; ns, not significant. The figures are representatives of two independent experiments.

To verify the migratory capacity of *Mid1^-/-^* T cells in EAE, fluorescently labeled *Wt* and *Mid1^-/-^* splenocytes were intravenously transferred into *Wt* EAE mice and their migration to the CNS, after 96 h, was determined by flow cytometry using single cell suspension prepared from both the spinal cord and peripheral lymphoid tissues (**Figure 7A**). There were similar proportions of labeled *Wt* and *Mid1^-/-^* DCs in the CNS of EAE mice (**Figures 7B-C**). In contrast, the *Mid1^-/-^* T cells were rarely seen in the CNS, but they were seen in abundance in peripheral tissue when compared to *Wt* (**Figures 7D-F**). We next reconstituted lymphocyte-deficient *Rag2^-/-^* mice (CD45.2) with a mixture of equal amounts of CD45.1-expressing *Wt* T cells and CD45.2-expressing* Mid1^-/-^* T cells, followed by EAE induction (**Figure 7G**). The chimeric mice with equal amounts of CD45.1-expressing *Wt* T cells and CD45.2-expressing* Mid1^-/-^* T cells successfully developed EAE after MOG_35-55_ immunization (**Figures 7H-I**). As shown in **Figure 7J**, the chimeric mice lacked CD45.1-expressing CD3^-^ cells, suggesting a high purity of enriched T cells. Flow cytometric detection of CNS-infiltrating cells after EAE induction showed that the proportion of CD45.2-expressing* Mid1^-/-^* T cells was much lower than that of CD45.1-expressing *Wt* T cells, while no significant difference in the percentage of CD45.1- and CD45.2-expressing cells was observed in the spleen (**Figures 7K-L**). This data indicates that Mid1 disruption suppressed the migration of T cells to the spinal cord.

### Midline-1 promotes T cell migration via mTOR signaling

**Figure 8:**
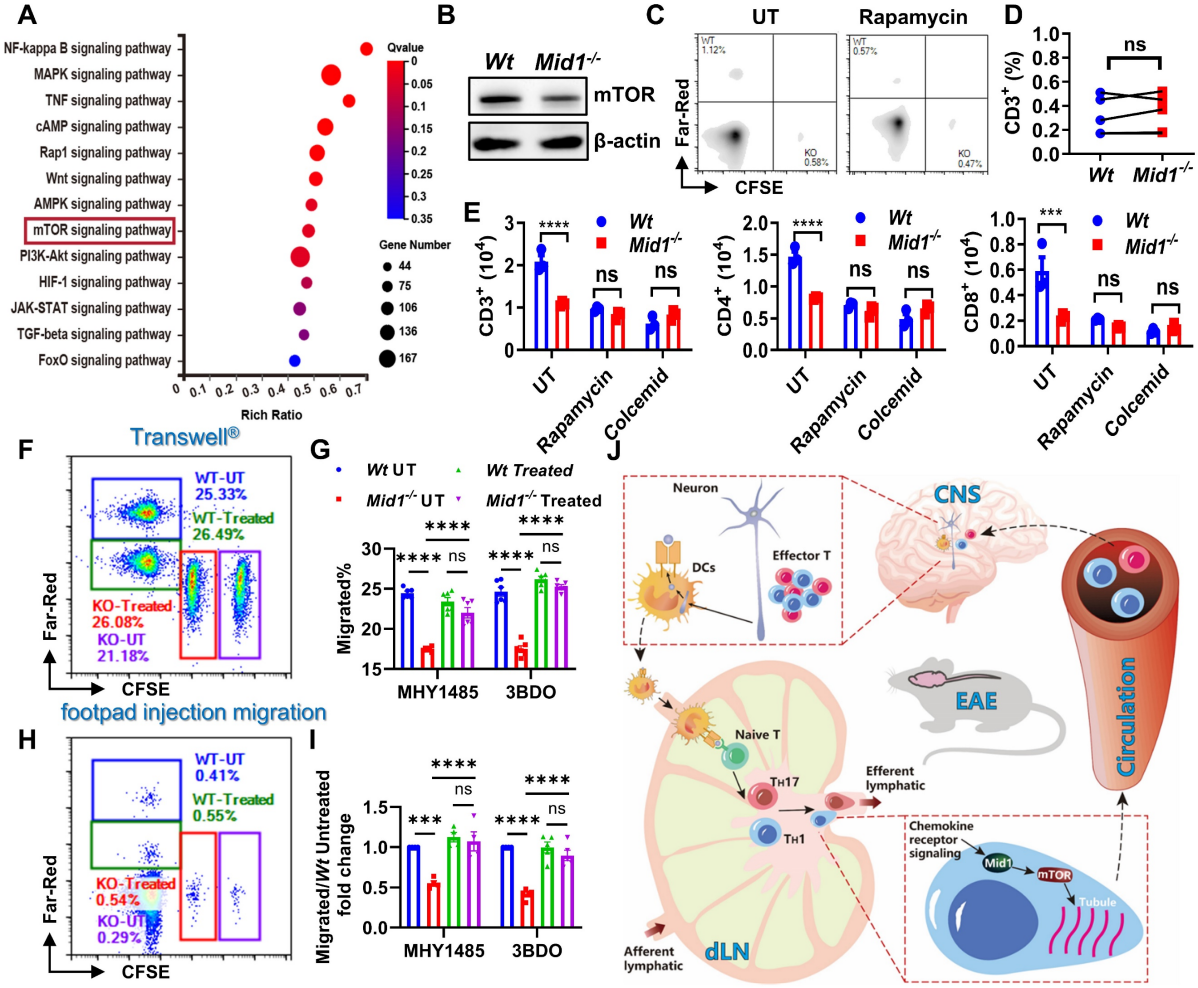
**Mid1 promotes T cell migration via mTOR signaling. A**, RNA-seq analysis was performed on spinal cord tissues from untreated *Wt* and *Mid1^-/-^* mice (n = 5), as well as *Wt* and *Mid1^-/-^* EAE mice (n = 4). KEGG pathway analyses of differentially expressed genes were performed to identify the most significantly enriched signal transduction pathways. **B**, T cells were isolated from splenocytes of *Wt* and *Mid1^-/-^* mice for western blot detection of mTOR. **C-D,** Splenocytes from normal *Wt* and *Mid1^-/-^* mice were pretreated with or without Rapamycin (3 μM) for 16 h and labeled with Far-Red and CFSE respectively. Cells were then mixed at a 1:1 ratio and injected into the footpad of *Wt* mice. After 12 h, the recipient mice were sacrificed and the fraction of Far-Red-labeled* Wt* and CFSE-labeled* Mid1^-/-^* T cells in the popliteal lymph nodes were measured by flow cytometry. Representative figures (**C**) and statistical analysis (**D**) were shown. ns, not significant. **E,** *Wt* and *Mid1^-/-^* splenocytes were pretreated with rapamycin (mTOR inhibitor) or colcemid (microtubule assembly inhibitor) for 24 h. The cells were then placed into the insert of a Transwell^®^ plate, with 400 ng/ml CCL-19 in the lower chamber. The cells that migrated into to the lower chamber were counted and staining with CD3, CD4, and CD8 antibodies after 6 h. ***, p < 0.001; ****, p < 0.0001; ns, not significant. **F-I**, *Wt* and *Mid1^-/-^* splenocytes were pretreated with mTOR activator MHY1485 (5 μM) or 3BDO (40 μM) for 6 h. Untreated *Wt* and *Mid1^-/-^* splenocytes were stained with 1 μM CellTrace™ Far-Red and 5 μM CFSE, whereas treated *Wt* and *Mid1^-/-^* splenocytes were labeled with 0.1 μM CellTrace™ Far-Red and 0.5 μM CellTrace™ CFSE respectively. Next, the four groups of cells labeled with distinct fluorescent dyes were mixed in equal quantities. **F-G**, The mixed cells were then seeded to the insert of a 24-well Transwell^®^ plate with 400 ng/mL CCL19 in the lower chamber. The cells migrated into the lower chamber were counted and analyzed by flow cytometry after 6 h of incubation in a 37 ℃ CO_2_ incubator. Representative figures (**F**) and statistical analysis (**G**) were shown. ***, p < 0.001; ****, p < 0.0001; ns, not significant. **H-I**, Mixed cells were subcutaneously injected into the footpad of *Wt* mice. After 12 h, the popliteal lymph nodes were isolated and the frequencies of CellTrace™ Far-Red and CFSE labeled CD3^+^ T cells were measured using flow cytometry. Representative figures (**H**) and statistical analysis (**I**) were shown. ****, p < 0.0001; ns, not significant. All figures are representatives of three independent experiments.** J**, Conclusion figure showing the mechanisms by which Mid1 regulates the migratory activity of effector T cells in EAE.

To investigate the potential pathways regulated by Mid1, we performed KEGG pathway enrichment analysis using differentially expressed genes of spinal cord tissues between *Wt* and *Mid1^-/-^* EAE mice. We identified multiple inflammation-related pathways, including the mTOR signaling pathway that is involved in cytoskeletal dynamics and migration^19, 27^ (**Figure 8A**). Furthermore, *Mid1*-deficient T cells showed a lower mTOR level than *Wt* T cells (**Figure 8B**). Treatment with rapamycin (an mTOR inhibitor) inhibited *in vivo* migration of* Wt* T cells, but had no obvious effect on *Mid1^-/-^* T cells (**Figures 8C-D**). Similarly, mTOR inhibition with rapamycin or suppression of microtubule polymerization with colcemid (a microtubule inhibitor) significantly suppressed the migratory ability of *Wt* T cells, but not *Mid1^-/-^* T cells in Transwell^®^ assay. A similar migratory capacity was observed in *Wt* and *Mid1^-/-^* T cells after inhibition of mTOR or microtubule polymerization (**Figure 8E**). Additionally, the Transwell^®^ assay following treatment of MHY1485 and 3BDO (mTOR agonist) showed that the agonist boosted the migration of *Mid1^-/-^* T cells, while it had no significant effect on *Wt* T cells (**Figure S9, Figures 8F-G**). The outcomes were subsequently confirmed *in vivo*, where MHY1485 and 3BDO therapy restored the migratory capacity of *Mid1^-/-^* T cells (**Figures 8H-I**). These results suggest that Mid1 regulates T cell migration via enhancing the mTOR/tubulin axis (**Figure 8J**).

## Discussion

Effector T cell activation, proinflammatory cytokine production, and T cell migration are crucial steps in regulating autoimmune response-mediated tissue damage. Several therapeutic approaches targeting T cell activation (*i.e.*, Janus kinase inhibitors) and proinflammatory cytokines (*i.e.*, antibodies against IL17, IFNγ, and TNFα) have been developed for the treatment of autoimmune disorders. However, there are no therapeutic agents targeting effector T cell migration, largely due to our limited understanding of regulatory mechanisms of T cell migration in autoimmune disease. In this study, we found that Mid1 is a key regulator of effector T cell migration from dLN to the CNS in EAE.

The role of Mid1 in autoimmune diseases is not clear. To the best of our knowledge, this is the first report illustrating the regulatory role of Mid1 in EAE and effector T cell migration towards the CNS. Mid1 is a microtubule-associated protein that is required for neuronal development. It suppresses axon growth and elongation, and thereby maintains the pattern of callosal projection in the cortex 28. Mid1 is highly conserved between humans and mice, and possesses ubiquitin E3 ligase activity 29. By binding to the α4 subunit of PP2A, Mid1 ubiquitinates and degrades PP2A 30, resulting in abnormal axonal development 28. Recent studies indicate that Mid1 is also expressed in airway epithelium and immune cells, thus playing an important role in asthma and viral infection 18, 23, 31. Via inactivating PP2A and suppressing distal TCR signaling, Mid1 has been shown to regulate degranulation and polarization of cytotoxic T cells 23, 32. However, it remains unclear as to the level that Mid1 is involved in autoimmune disease.

In the current study, we found that Mid1 was upregulated in the CNS of EAE mice and its deficiency alleviated disease severity and inflammatory infiltration in both active and passive EAE. During EAE development, myelin antigens are first processed and presented by antigen-presenting cells such as DCs. Activated DCs then migrate to dLN where they present the antigenic peptides to T lymphocytes, leading to the activation and differentiation. The activated effector T cells subsequently migrated to the CNS, mediating neuronal destruction 6. RNA sequencing, flow cytometry, histology, and immunofluorescence data suggest that Mid1 deletion reduced effector T cell infiltration in the CNS. *Rag2^-/-^* mice reconstituted with *Mid1^-/-^* T cells exhibited less severe symptoms of EAE and had a reduced number of T cells in the CNS after MOG_35-55_ induction. Transcriptomic analysis of spinal cord tissues also revealed that differentially expressed genes between* Wt* and *Mid1^-/-^* were mainly enriched in T cell-related pathways. These results demonstrate that Mid1 primarily regulates EAE via T cells. Unlike with T cells, we did not find significant differences in DC numbers between* Wt* and *Mid1^-/-^* mice after EAE induction.

In contrast to the elevated effector T cells in the dLN of EAE mice compared with controls, we found that the proportions and quantities of T cells and effector T cells in the dLN of *Mid1^-/-^* mice were significantly higher in both active and passive EAE models. In addition, co-transfer experiment of labeled *Wt* and *Mid1^-/-^* T cells into EAE animals demonstrated much less infiltration of *Mid1^-/-^* T cells in the CNS. This suggests that Mid1 may affect the migration of T cells from the dLN to the CNS. Therefore, we investigated if the defect of Mid1 may affect T cell migration in both *in vitro* and *in vivo* experiments. Transwell^®^ *in vitro* migration assay validated the hypothesis that *Mid1^-/-^* T cells have a reduced migratory ability. By observing the *in vivo* migration of CellTrace™ Far-Red-labeled *Wt* and CFSE-labeled *Mid1^-/-^* splenic T cells from the footpad to popliteal lymph nodes, we also found a much smaller amount of CFSE-labeled *Mid1^-/-^* T cells migrated to the popliteal lymph nodes. To further elucidate T cell migration in the pathological condition of EAE, a mixture of fluorescently labeled *Wt* and *Mid1^-/-^* splenocytes were co-transferred into EAE mice. Similarly, fewer Far-Red-labeled* Mid1^-/-^* T cells migrated to the CNS of EAE recipients compared to* Wt* T cells. In contrast, a higher proportion of *Mid1^-/-^* T cells were detected in the spleen and dLN. In a subsequent experiment, CD45.1/CD45.2 chimeric mice were constructed by co-transfer of equal amounts of *CD45.1^+^ Wt* and *CD45.2^+^ Mid1^-/-^* T cells into *Rag2^-/-^* mice, followed by MOG_35-55_ immunization. Higher numbers of *CD45.1^+^ Wt* T cells were found in the CNS of EAE mice. Based on this data, we were able to determine that Mid1 is critical for T cell migration in EAE.

In addition to T cell migration, T cell differentiation and proliferation also play a key role in the pathogenesis of EAE 26, 27, 33. We did not find a significant impact of Mid1 deletion on T cell differentiation to T_H_1 and T_H_17 in EAE as evidenced by similar percentages of effector T_H_1 and T_H_17 in the CNS between *Wt* and *Mid1^-/-^* mice after EAE induction. *Wt* and *Mid1^-/-^* T cells also showed a similar capacity to polarize to T_H_1 and T_H_17 subsets, as well as a similar level of proliferation and activation in *in vitro* differentiation studies.

Cytoskeletal rearrangement plays a crucial role in cell migration 34 and we have reported that Mid1 deletion results in disrupted cytoskeletal rearrangement and migration 24. However, the mechanism via which Mid1 regulates cytoskeletal rearrangement and cell migration is unknown. mTOR has been reported to regulate cytoskeletal dynamics and migration 35, 36 and herein we observed a reduction of the mTOR signaling pathway in *Mid1^-/-^* mice after EAE induction. We further demonstrated that Mid1 deletion leads to a reduced expression of mTOR and suppression of mTOR abolished Mid1-induced T cell migration in *Wt* T cells. In addition, mTOR agonists restored the migratory ability of *Mid1^-/-^* T cells. Mid1 has been reported to ubiquitinate PP2A and mediate its degradation 22, while PP2A, as a negative regulator of mTOR, reduces mTOR signaling 37. Therefore, we examined if Mid1-mediated T cell migration is dependent on mTOR and found that both mTOR blockade with rapamycin and microtubule inhibition with colcemid abolished Mid1-associated T cell migration. These results were further validated *in vivo* by footpad injection migration assay. Therefore, Mid1 may regulate T cell migration by upregulating mTOR signaling.

In summary, the present study suggests that Mid1 deletion can alleviate EAE by inhibiting mTOR-dependent T-cell migration from lymph nodes to CNS. There are several limitations of this study. First, the regulatory effect of Mid1 on other types of cells was not examined in this study as we focused on the regulatory role of Mid1 in T cell migration. The involvement of other cell types such as dendritic cells and microglia was not studied. Second, we did not utilize human tissue samples to verify the effect of Mid1 on MS. Although Mid1 is highly conserved between humans and mice, the regulatory role of Mid1 in human MS requires further investigation. Lastly, the mechanism by which Mid1 affects the mTOR pathway requires further validation. Although PP2A has been reported to mediate Mid1-induced mTOR activation 37, it is not clear if other mechanisms, such as direct ubiquitination by Mid1, are involved in Mid1-induced mTOR signaling.

## Supplementary Material

Supplementary materials and methods, figures.

## Abbreviations

CNS: central nervous system
DCs: dendritic cells
DLNs: draining lymph nodes
EAE: experimental autoimmune encephalomyelitis
IF: immunofluorescence
MFI: mean fluorescence intensity
Mid1: midline-1
MS: multiple sclerosis
PCA: principal component analysis
PTX: pertussis toxin
SEM: standard error of the mean
TRIM: triple-motif family
VCAM-1: type I vascular cell adhesion proteins
