# Supplementary material for: Midline-1 regulates effector T cell motility in experimental autoimmune encephalomyelitis via mTOR/microtubule pathway
Source: Theranostics. 2024 Jan 20;14(3):1168–80. doi: 10.7150/thno.87130 (PMC10845203; doi:10.7150/thno.87130)
Supplement: Supplementary file 1 — Supplementary materials and methods, figures. [file thnov14p1168s1.pdf]

SUPPLEMENTARY MATERIALS AND METHODS

Mice

Mid1$^{-/-}$ mice were generated using CRISPR-Cas9 technology as we previously described [1]. *Rag2*$^{-/-}$ and *CD45.1* mice were purchased from Shanghai Model Organisms Center, Inc. All the mice used in the experiment were kept in the SPF animal facilities at Tongji Hospital. All experiments reported herein were approved by the Institutional Animal Care and Use Committee of Tongji Hospital and conducted in accordance with the animal use guidelines of the institute.

Induction and evaluation of active EAE

For the construction of chimeric mice, *Rag2*$^{-/-}$ mice were adoptively transferred with 10^7 wild-type (*Wt*) T cells, *Mid1*$^{-/-}$ T cells, or a mixture of CD45.1-expressing *Wt* and CD45.2-expressing *Mid1*$^{-/-}$ T cells at 1:1 ratio. *Wt*, *Mid1*$^{-/-}$, or chimeric mice were then used for the induction of active EAE as previously reported[2]. Briefly, mice were subcutaneously injected with a 200 μL emulsion containing 1 mg/ml MOG_{35-55} peptide (MEVGWYRSPFSRVVHLYRNGK), 2.5 mg/ml Mycobacteria tuberculosis H37Ra (B.D.), and 100 μL Freund's adjuvant (Sigma). On the day of immunization and two days after immunization, mice were intraperitoneally injected with 200 ng of pertussis toxin (List Biological Laboratories Inc.) to improve the efficiency of EAE induction[3]. EAE was scored as follows: 0 – normal; 1 – paralyzed tail; 2 – moderate hind limb weakness; 2.5 – one hind limb paralyzed; 3 – both hind limbs paralyzed; 3.5 – hind limbs paralyzed and forelimbs weakness; 4 – forelimbs paralyzed; and 5 – moribund.

Induction of passive EAE

For the induction of passive EAE, pathogenic lymphocytes were isolated from draining lymph nodes and spleen of MOG_{35-55}-immunized *Wt* or *Mid1*$^{-/-}$ mice immediately after disease onset. Cells were then cultured at the concentration of 10^7/mL in the presence of 20 μM MOG_{35-55} and 20 ng/mL IL-2 (PeproTech) for 48 h. Cultured cells (5 x 10^6/mouse) were intravenously injected into recipient mice via angular vein to induce transfer EAE as described earlier [4].

Isolation of spinal cord-infiltrating inflammatory cells

For single cell isolation, murine spinal cord tissue was cut into small pieces, then digested with 1 mg/ml type II collagenase (ThermoFisher Scientific) at 37 ℃ in a 150-rpm shaking water bath for 40 min. The resulting cells were resuspended in 40% Percoll and carefully added onto a layer of 70% Percoll and centrifuged at 500 x g to remove histiocytes and erythrocytes. The immune cells were collected from the cloudy layer at the interface between the two Percoll layers. These cells were washed with 1 x PBS buffer and utilized in the following studies.

T cells differentiation

For T cells activation, splenic single-cell suspensions were stimulated with 1 μg/mL anti-CD3 (clone# 145-2C11, Biolegend) and 1 μg/mL anti-CD28 (clone# 37.51, Biolegend) for 72 h. For T_H1/T_C1 polarization, T cells were stimulated with 1 μg/mL anti-CD3 and 1 μg/mL anti-CD28 in the presence of 20 ng/ml IL-12 (PeproTech). For T_H17/T_C17 polarization, T cells were stimulated with 1 μg/mL anti-CD3 and 1 μg/mL anti-CD28 in the presence of 2.5 ng/mL TGF-β (Biolegend) and 100 ng/mL IL-6 (PeproTech). All cells were cultured in RPMI-1640 medium supplemented with 10% fetal bovine serum (Gibco) and 5 ng/mL recombinant murine IL-2 (PeproTech).

Transwell®

Splenocytes isolated from *Wt* and *Mid1*$^{-/-}$ mice were treated with or without the presence of 3 μM rapamycin (MCE) or 100 ng/mL colcemid (Acmec biochemical) for 18 h, and then placed in the insert of a Transwell® plate, with the lower chamber filled with 600 μL RPMI-1640 medium supplemented with 10% fetal bovine serum and 400 ng/mL C-C motif chemokine ligand 19 (CCL19, Biolegend). After 6 of incubation in a 37 ℃ CO_2 incubator, cells were collected from the lower and upper chambers for cell counting and flow cytometric detection after staining with anti-mouse CD3, CD4, and CD8 antibodies, as previously described [1]. For Transwell® assay with mTOR activator treatment, *Wt* and *Mid1*$^{-/-}$ splenocytes were pretreated with either 5 μM MHY1485 (MCE) or 40 μM 3BDO (MCE) for 6 h. Untreated *Wt* and *Mid1*$^{-/-}$ splenocytes were stained with CellTrace™ Far-Red and CFSE, whereas treated *Wt* and *Mid1*$^{-/-}$ splenocytes were labeled with 0.1 μM

CellTrace™ Far-Red and 0.5 μM CellTrace™ CFSE respectively. Next, the four groups of cells with distinct fluorescent staining were mixed in equal amounts. The cells were placed into the insert of a 24-well Transwell® plate, with the lower chamber filled with RPMI-1640 medium containing 400 ng/mL CCL-19. The cells migrated into the lower chamber were counted and analyzed by flow cytometry after 6 h of incubation in a 37 ℃ CO_2 incubator.

In vivo T cells migration

Splenic T cells from *Wt* and *Mid1*$^{-/-}$ mice with or without pretreatment of rapamycin (3 μM, MCE) for 18 h were labeled with CellTrace™ Far-Red or CellTrace™ CFSE (ThermoFisher Scientific). Labeled cells were then mixed at a 1:1 ratio and injected into the footpad of *Wt* mice. For the treatment of mTOR agonists, MHY1485 (5 μM, MCE) or 3BDO (40 μM, MCE) was added to *Wt* and *Mid1*$^{-/-}$ splenocytes for 6 h. Untreated *Wt* and *Mid1*$^{-/-}$ cells were fluorescently labeled with CellTrace™ Far-Red (1 μM) and CellTrace™ CFSE (5 μM) respectively, while mTOR agonist-treated *Wt* and *Mid1*$^{-/-}$ splenic cells were labeled with 0.1 μM CellTrace™ Far-Red and 0.5 μM CellTrace™ CFSE respectively. The four groups of cells were then mixed in equal proportions and injected into the footpad of *Wt* mice. Popliteal lymph nodes were harvested 12 h after injection and the percentages of CellTrace™ Far-Red and CFSE-labeled CD3$^+$ cells were then measured via flow cytometry. For *in vivo* T cell migration in the scenario of EAE, fluorescently labeled splenic cells as described above were adoptively transferred to EAE mice. The spinal cord, draining lymph nodes, and spleen were collected for the isolation of single cell suspension after 96 h, and the ratio of CellTrace™ Far-Red and CFSE-labeled CD3$^+$ cells was quantified by flow cytometry.

Histology

Histological examinations were used to assess immune infiltration and demyelination. Spinal cords isolated from mice were embedded in paraffin after fixation. Immune infiltrations were assessed by H&E staining (Solarbio) and demyelination was examined by Luxol Fast Blue (LFB, Solarbio) staining of spinal cord sections as we previously described [2].

Immunofluorescence

Spinal cord sections were incubated with anti-Mid1 (1:200, Boster) and anti-CD3 (1:200, Abclonal) overnight at 4 °C, followed by staining with AF488-conjugated goat anti-rat IgG antibody (1:200, Yeasen) after being washed with 1 x PBST buffer. Nuclear staining was performed using DAPI (Solarbio). The pictures were taken at the magnification of x200.

Cell staining

Cells were stimulated with Cell Activation Cocktail (Biolegend) for 4.5 h, followed by cell surface marker staining with indicated antibodies. Cells were then fixed and permeabilized in fixation and permeabilization buffer (Invitrogen) for 30 min, followed by staining with anti-cytokine antibodies in permeabilization buffer (Biolegend) at room temperature for 1 h. All antibodies used for flow cytometry were purchased from Biolegend or BD Biosciences. Flow cytometric detection was performed on a CytoFlex cytometer (Beckman) or ACEA NovoCyte cytometer (Agilent Technologies) and analyzed using NovoExpress software.

Cytometric Bead Array (CBA)

Plasma cytokine concentrations (TNFα, IFN-γ, IL-6, and IL-10) were determined by CBA Mouse Inflammation Kit (BD Biosciences) as instructed by the manufacturer.

RT-PCR

Total RNA was extracted from the spinal cord by TRIzol Reagent (Takara) as instructed by the manufacturer. Reverse transcription of total RNA was performed using cDNA Transcriptase kit (Yeasen). The real-time PCR amplification of target genes was performed on a Quantage225 thermal cycler (Kubo Technology). The primer sequences for *Gapdh*, *Mid1*, and *Cd3* genes are listed below. *Gapdh, forward: GGT TGT CTC CTG CGA CTT CA; reverse: TGG TCC AGG GTT TCT TAC TCC; Mid1, forward: GGA GCT GAC CTG TCC TAT TTG; reverse: CCG CTC ACA GAT GCT TTC T. Cd3, forward: TGG AGC AAG AAT AGG AAG GC; reverse: CAT AGT CTG GGT TGG GAA CAG.*

Western blot

RIPA (Yeasen) was used to extract the total proteins from mouse splenic T cells, which were subsequently separated on SDS-PAGE and transferred to a PVDF membrane (0.45 m pore size, Millipore). The membranes were blocked using a 5% skimmed milk in TBST (TBS buffer containing 0.05% TWEEN). Next, the membranes were incubated with the indicated primary antibody overnight at 4°C, followed by 5 times of washes with TBST buffer and 1 h incubation with secondary antibody (1:5000, Cell Signaling Technology) at room temperature. For primary antibodies, anti-Mid1 antibody (1:1000, abcam) and anti-β-actin antibody (1:2000, Cell Signaling Technology) were used.

RNA-Sequencing

For RNA sequencing, total RNA was sent to BGI Genomics for sequencing and analyzed using the Dr.Tom system.

Statistical analysis

All data are presented as mean ± standard error of the mean (SEM). The difference in EAE clinical score was assessed with two-way ANOVA and the EAE incidence was compared with χ^2 test using GraphPad Prism 8. The differences between the means of two groups were accomplished by unpaired Student's t-test. The differences between the means of three or more independent groups were accomplished by one-way ANOVA. Using GraphPad Prism 8 for statistical analysis and graphing, $p < 0.05$ was considered statistically significant.

References

1. Rao X, Razavi M, Mihai G, Wei Y, Braunstein Z, Frieman MB, et al. Dipeptidyl Peptidase 4/Midline-1 Axis Promotes T Lymphocyte Motility in Atherosclerosis. Advanced science (Weinheim, Baden-Wurttemberg, Germany). 2023: e2204194.
2. Zhong J, Yu Q, Yang P, Rao X, He L, Fang J, et al. MBD2 regulates TH17 differentiation and experimental autoimmune encephalomyelitis by controlling the homeostasis of T-bet/Hlx axis. J Autoimmun. 2014; 53: 95-104.
3. Stromnes IM, Goverman JM. Active induction of experimental allergic encephalomyelitis. Nat Protoc. 2006; 1: 1810-9.
4. Stromnes IM, Goverman JM. Passive induction of experimental allergic encephalomyelitis. Nat Protoc. 2006; 1: 1952-60.

SUPPLEMENTARY FIGURES

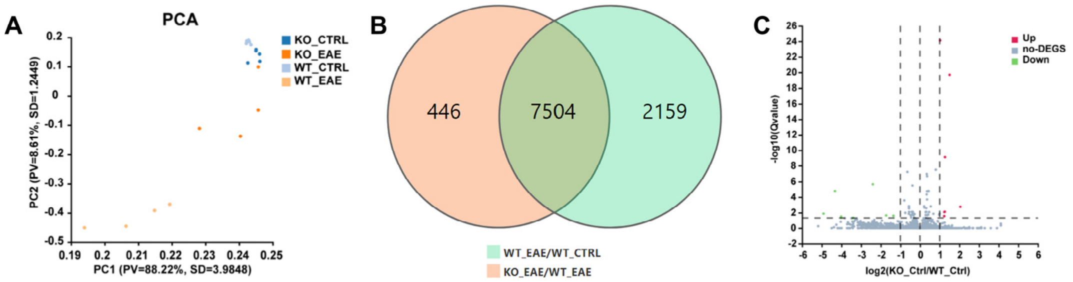

Figure S1: A. The principal component analysis (PCA) of the spinal cord RNA sequencing data of untreated or MOG35-55-immunized *Wt* and *Mid1*$^{-/-}$ mice (n = 5 for untreated and n = 4 for EAE mice). **B.** Venn diagram showing the logical relations between differentially expressed gene sets of KO_EAE *vs*. WT_EAE and WT_EAE *vs*. WT_Ctrl. **C.** Volcano plot showing differentially expressed genes for KO_Ctrl *vs*. WT_Ctrl.

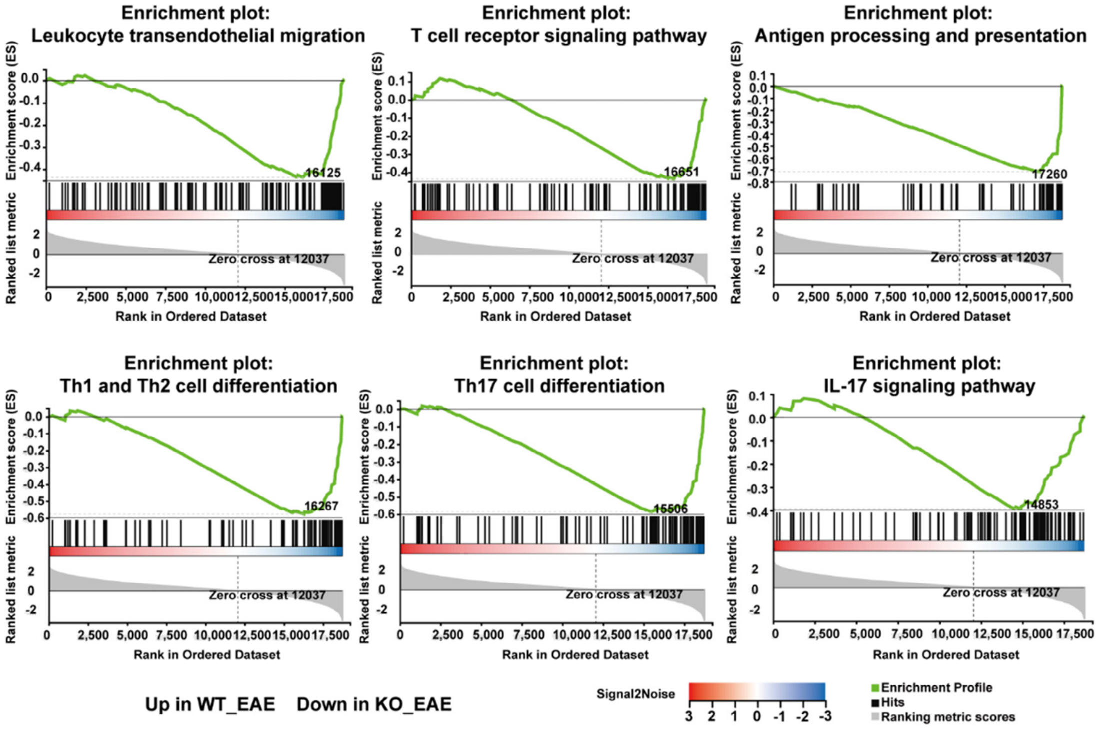

Figure S2: RNA-seq analysis was performed using the spinal cord tissues collected from untreated or MOG35-55-immunized *Wt* and *Mid1*$^{-/-}$ mice (n = 5 for untreated and n = 4 for EAE mice). GSEA enrichment analysis was carried out to show the alterations in T cell-related in WT_EAE and Mid1_EAE groups.

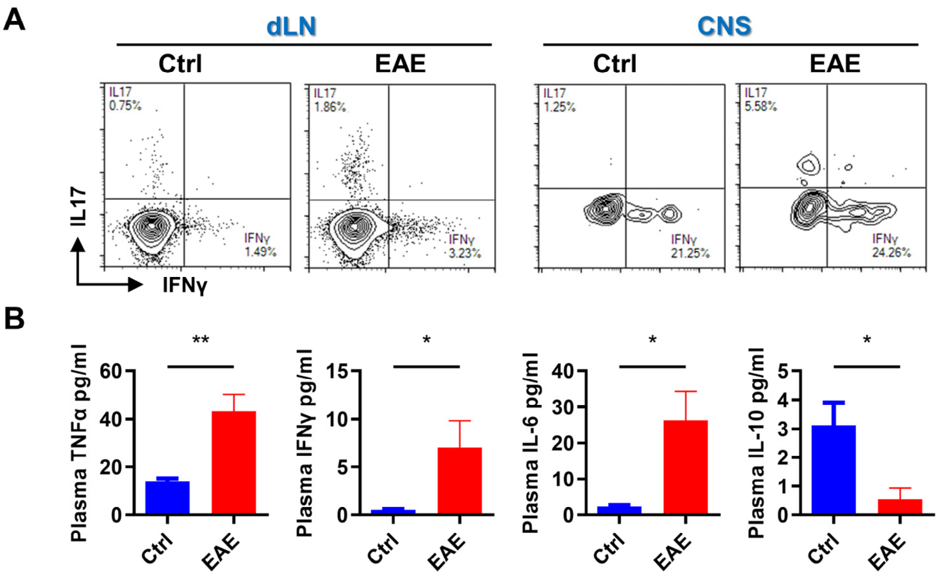

Figure S3: **A,** Obtained the cells from the control and EAE mice spinal cord and draining lymph nodes. Single-cells were stained with anti-mouse CD45, CD3, IFNγ and IL17, and flow cytometry assays were performed. This figure showed a representative density map of $CD45^{+}IFN\gamma^{+}$, $CD45^{+}IL17^{+}$ cells in CNS, and $CD3^{+}IFN\gamma^{+}$, $CD3^{+}IL17^{+}$ cells in draining lymph nodes. **B,** The plasma samples were collected for Cytometric Bead Array (CBA) detection of TNFα, IFN-γ, IL-6, IL-10. *, $p < 0.05$; **, $p < 0.01$.

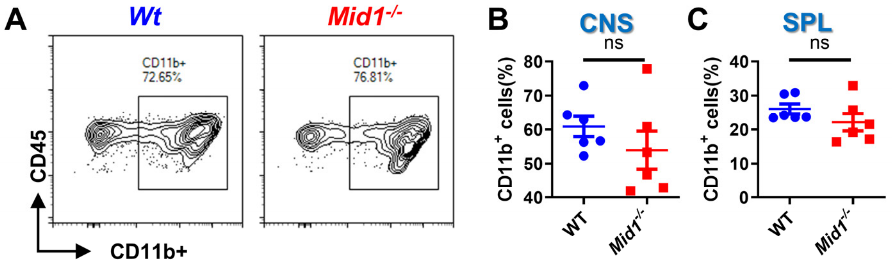

Figure S4: Single cells from the spinal cord or spleen of EAE mice were stained with anti-mouse CD45 and CD11b antibodies. Representative flow cytometric density plots (**A**) and statistical bar graphs (**B**) showed the proportions of $CD11b^{+}$ in the spinal cord of *Wt* and *Mid1*$^{-/-}$ mice. Data are shown as mean ± SEM. ns, not significant. **C**, Bar graph showing the proportion of $CD11b^{+}$ in the spleen of *Wt*/*Mid1*$^{-/-}$ EAE mice. Data are shown as mean ± SEM. ns, not significant.

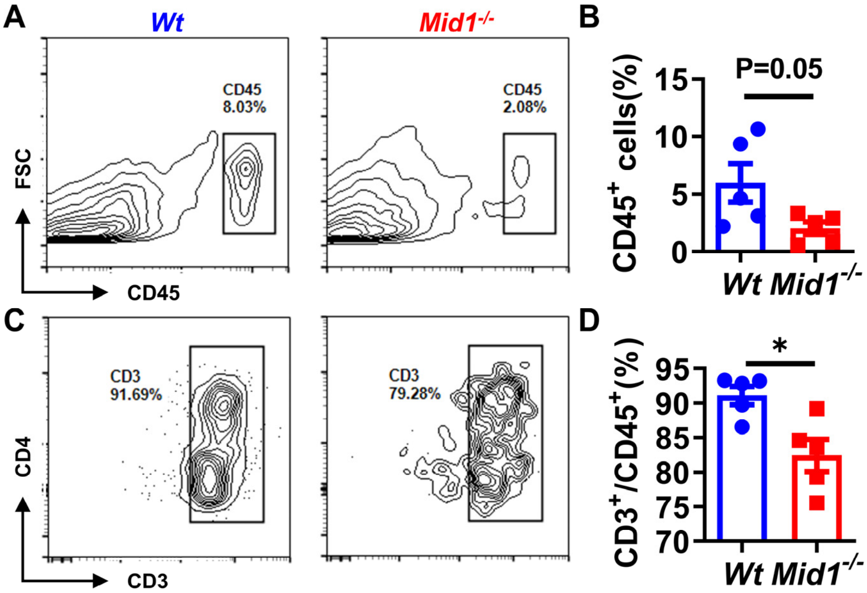

Figure S5: Spinal cord tissues were isolated for the preparation of single cell suspension 1 week after EAE onset. Cells were then stained with anti-mouse CD45, CD3, and CD4, Representative images showing the gating of $CD45^+$ (**A**) and $CD3^+$ T cells (**C**). The proportions of $CD45^+$(**B**), and $CD45^+CD3^+$ T cells (**D**) were analyzed. Data are shown as mean ± SEM. *, $p < 0.05$.

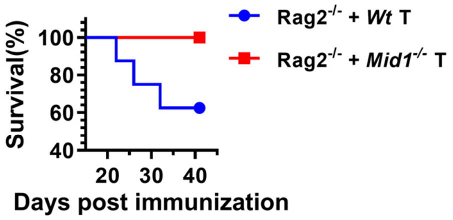

Figure S6: *Wt* and *Mid1$^{-/-}$* T cells were harvested from *Wt* and *Mid1$^{-/-}$* mice and were transferred to *Rag2$^{-/-}$* mice to generate chimeric mice with *Wt* or *Mid1$^{-/-}$* T cells. EAE was then induced with MOG_{35-55} 48 h later. The mortality of mice was observed.

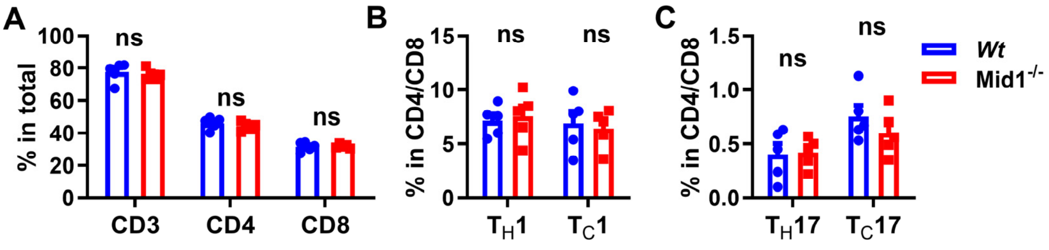

Figure S7: A, Single-cell suspensions were prepared from the lymph nodes of untreated *Wt* and *Mid1*$^{-/-}$ mice (10-12 weeks old). The proportions of CD3, CD4, and CD8 T cells were determined by flow cytometry. Data are shown as mean ± SEM. **B—C,** The proportions of TH1, TH17, TC1, and TC17 were determined. Data are shown as mean ± SEM. ns, not significant.

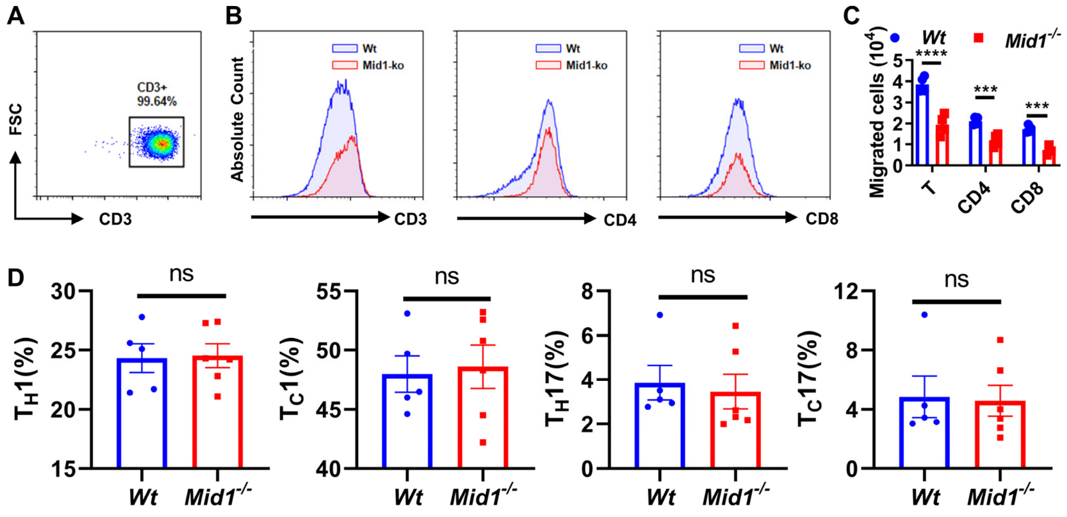

Figure S8: A, T cells were purified from the spleen of *Wt* and *Mid1*$^{-/-}$ mice using a mouse T cell enrichment kit. Flow cytometry showed the purity of the T cells is over 99%. **B—C,** Isolated *Wt* and *Mid1*$^{-/-}$ T cells were placed into the insert of a 24-well Transwell® plate, with the lower chamber filled with RPMI-1640 medium containing 400 ng/mL mouse recombinant CCL-19. After 6 h of incubation in a 37°C CO_2 incubator, the cells migrated to the lower chamber were counted and harvested for flow cytometric detection of $CD3^+$, $CD4^+$, and $CD8^+$ cells. Representative histograms (**B**) and statistical analysis (**C**) showed the migration of *Wt* and *Mid1*$^{-/-}$ $CD3^+$, $CD4^+$, and $CD8^+$ T cells. ***, $p < 0.001$; ****, $p < 0.0001$. **D,** Purified *Wt* and *Mid1*$^{-/-}$ T cells were cultured under the differentiation conditions of TH1 (anti-CD3, anti-CD28, IL-12) and TH17 (anti-CD3, anti-CD28, TGFβ, IL-6) for 72 h, followed by intracellular flow cytometric detection of IFNγ and IL17 expressions. The proportions of TH1 ($CD4^+$ $IFN\gamma^+$), TH17 ($CD4^+$ $IL17^+$), TC1 ($CD8^+$ $IFN\gamma^+$), and TC17 ($CD8^+$ $IL17^+$) were determined. Data are shown as mean ± SEM. ns, not significant. The figures are representatives of two independent experiments.

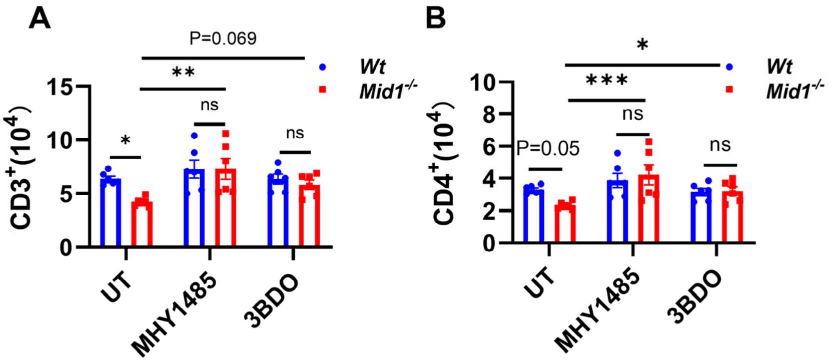

Figure S9: *Wt* and *Mid1*$^{-/-}$ splenic cells were treated with or without the presence of 5 μM MHY1485 (MCE) or 40 μM 3BDO (MCE) for 6 h, the cells were added to the insert of a 24-well Transwell® plate, with the lower chamber filling with 1640 medium containing 400 ng/mL CCL-19. The cells migrated to the lower chamber were counted and stained with CD3, CD4 and CD8 antibodies for flow cytometric analysis after 6 h. *, $p < 0.05$; **, $p < 0.01$; ***, $p < 0.001$; ns, not significant.
